# *Breaking Bad*: Autophagy Tweaks the Interplay Between Glioma and the Tumor Immune Microenvironment

**DOI:** 10.3389/fimmu.2021.746621

**Published:** 2021-10-04

**Authors:** Yuxiang Fan, Yubo Wang, Jian Zhang, Xuechao Dong, Pu Gao, Kai Liu, Chengyuan Ma, Gang Zhao

**Affiliations:** Department of Neurosurgery, The First Hospital of Jilin University, Changchun, China

**Keywords:** autophagy, glioma, MDSC, neutrophil, tumor-associated macrophage, tumor immune microenvironment

## Abstract

Though significant strides in tumorigenic comprehension and therapy modality have been witnessed over the past decades, glioma remains one of the most common and malignant brain tumors characterized by recurrence, dismal prognosis, and therapy resistance. Immunotherapy advance holds promise in glioma recently. However, the efficacy of immunotherapy varies among individuals with glioma, which drives researchers to consider the modest levels of immunity in the central nervous system, as well as the immunosuppressive tumor immune microenvironment (TIME). Considering the highly conserved property for sustaining energy homeostasis in mammalian cells and repeatedly reported links in malignancy and drug resistance, autophagy is determined as a cutting angle to elucidate the relations between glioma and the TIME. In this review, heterogeneity of TIME in glioma is outlined along with the reciprocal impacts between them. In addition, controversies on whether autophagy behaves cytoprotectively or cytotoxically in cancers are covered. How autophagy collapses from its homeostasis and aids glioma malignancy, which may depend on the cell type and the cellular context such as reactive oxygen species (ROS) and adenosine triphosphate (ATP) level, are briefly discussed. The consecutive application of autophagy inducers and inhibitors may improve the drug resistance in glioma after overtreatments. It also highlights that autophagy plays a pivotal part in modulating glioma and the TIME, respectively, and the intricate interactions among them. Specifically, autophagy is manipulated by either glioma or tumor-associated macrophages to conform one side to the other through exosomal microRNAs and thereby adjust the interactions. Given that some of the crosstalk between glioma and the TIME highly depend on the autophagy process or autophagic components, there are interconnections influenced by the status and well-being of cells presumably associated with autophagic flux. By updating the most recent knowledge concerning glioma and the TIME from an autophagic perspective enhances comprehension and inspires more applicable and effective strategies targeting TIME while harnessing autophagy collaboratively against cancer.

## Introduction

Gliomas originate from supportive glial cells in people with dichotomy prognosis and limited treatment responses (1). Generally, its prognosis depends on the pathologic grade and genetic mutation profile, whose level of concern is checked through isocitrate dehydrogenase (IDH) and *1p/19q* status (2). Lower-grade glioma (LGG, WHO II-III grade) with prognostically favorable mutations yields the most benefits from multimodality approaches like early surgical resection, radiotherapies, chemotherapies, and other anti-tumor comprehensive therapies, while it remains a challenge to extend survival for other malignant types (3). The median survival time of patients with glioblastoma multiforme (GBM, WHO IV grade) is merely 14 months and the H3 Lys27Met-mutant glioma holds the worst prognosis: a 2-year survival rate less than 10%, among all diffuse gliomas (4). Recent advances have been made in exploring potential therapies by targeting the tumor immune microenvironment (TIME) in glioma.

As immunotherapies prevail in cancers, the limited responses in glioma to treatment lead to a reexamination of the core of immunotherapy: the infiltrating immunocytes and their local microenvironment. Immune infiltration in glioma through the disrupted blood-brain barrier (BBB) deprives the central nervous system (CNS) of “immune privilege” - restrictive entry of circulatory immune cells (5, 6). It is reported to be pertinent to glioma oncogenesis, progression, and therapy resistance (7, 8). The infiltrative immune cells, including tumor-associated macrophages/microglia (TAM), myeloid-derived suppressor cells (MDSCs), dendritic cells (DCs), neutrophils, and tumor-infiltrating lymphocytes, are meant to maintain intercellular homeostasis by eliminating abnormalities though the initial targets which ultimately somehow compromise (5, 9). Together with a few exhausted T cells, nonfunctional natural killer cells (NK cells), inflammatory mast cells, cancer-associated fibroblasts, diffusely distributed astrocytes, immunosuppressive cytokines, insufficient nutrient supply, and hypoxia, the glioma immune microenvironment is roughly characterized (10).

The immune microenvironment plays a dual role in glioma. Both innate and adaptive immune responses exert influence to retain control of glioma, whilst glioma inversely manipulates immune cells to attain immune suppression and evasion (11). It warrants more studies unraveling the potential mechanisms that glioma utilizes to shift functional immune cells towards being tumorigenic. Thus, it becomes possible to restore immune efficacy and revive the success of immunotherapies. Specifically, one way that could be employed not only by glioma but also by immune cells to adapt to both intrinsic and extrinsic alterations is autophagy.

Autophagy ensures cellular homeostasis and recycles cytoplasmic entities for energy supply when under stress (1). It typically includes three primary subtypes: macroautophagy, microautophagy, and chaperone-mediated autophagy (CMA). Despite the three morphologically unique forms, they all end up in the degradation of targets within lysosomes consonantly (12). In brief, macroautophagy, widely known as autophagy, uses autophagy adaptor proteins like p62/SQSTM1 to label cytoplasmic cargo for a double-membrane vesicle called autophagosome and lysosome degradation (13). In contrast, microautophagy directly encapsulates cellular cargos with endosomal membranes or invagination of lysosomal. CMA is characterized by the chaperone-binding cargos with Lys-Phe-Glu-Arg-Gln (KFERQ) -like pentapeptide motif entering lysosomes *via* lysosomal-associated membrane protein 2a (LAMP2a) (14). The detailed autophagy phases and machinery for each subtype are beyond the scope of this review and have already been extensively reviewed (15).

A myriad of evidence show that autophagy is exploited by glioma to resist therapies and by immune cells to dampen anti-tumor responses (16, 17). A study manifests that it is autophagy that is blocked by chloroquine (CQ), thereby enhancing cytotoxicity of temozolomide (TMZ) to glioma cells (18). Additionally, by analogy to the mammalian target of rapamycin (mTOR) inhibitor rapamycin, indoleamine 2,3-dioxygenase (IDO) -mediated tryptophan depletion educates T cells towards immune tolerance through triggering autophagy (19). Concerning the complex nature, it might be more informative and illuminating to integrate into a summary with the updated works of literature relative to autophagy in the glioma immune microenvironment.

In this review, the heterogeneity of tumor immune microenvironment in glioma is discussed, along with the reciprocal impacts on both sides. It also unveils the way that autophagy aids malignancy by switching itself between a cytoprotective role and a cytotoxic one within glioma. The fact that autophagy plays a pivotal part in modulating glioma cells and the members in the TIME, and thereby influencing the subtle interactions among the components of the microenvironment, are specifically highlighted. Overall, this review aims to pave the way for a resounding success of immunotherapies adjuvant with autophagy modulators for glioma in the near future.

## The Interactive Tumor Immune Microenvironment in Glioma

**Figure 1:**
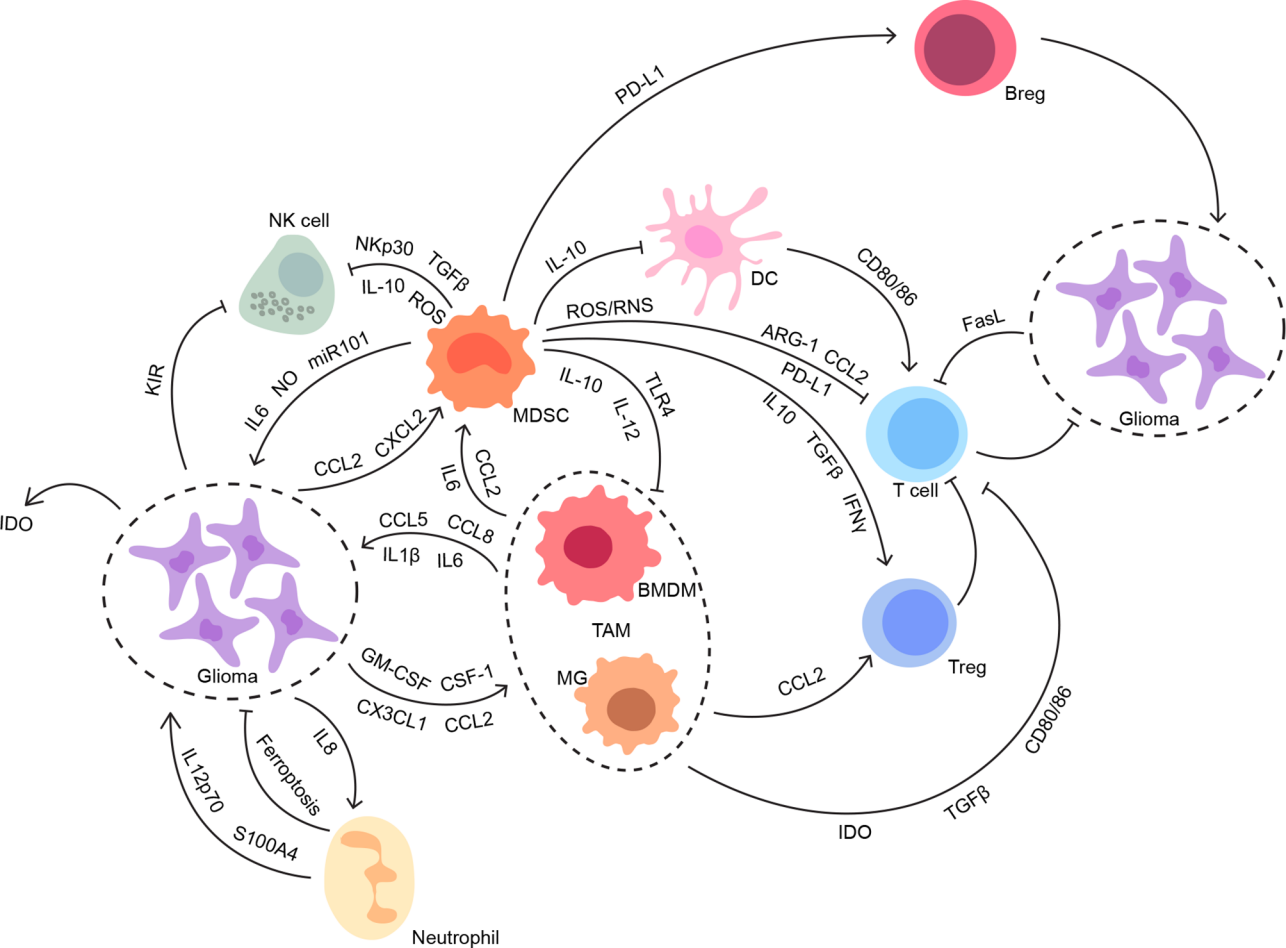
The interconnections between glioma and its immune microenvironment. Both innate and adaptive immune systems contribute to the suppressive immune microenvironment in glioma. The intricate interconnections among the members of the glioma immune microenvironment work synergistically to facilitate tumor progression without disturbance, especially from anti-tumor immunity.

Despite a paucity of immune cells and limited lymphatic drainage, immunosurveillance within a healthy brain with brain-specific microglia is capable of stimulating a modest and highly regulated immune response (20). Circulating immune cells should have infiltrated in the CNS across the impaired BBB in the presence of glioma (21). Still, glioma displays a “cold tumor” phenotype with a low number of immunogenic effector immune cells compared with other tumors, which might be related to the limited efficacy of immunotherapies (22). Given the paradox of immune responses before and after glioma development, the intricate regulations of the immune microenvironment involved with both cellular and molecular mechanisms deserve more attention and discussion (**Figure 1**).

### Tumor-Associated Macrophage/Microglia

TAMs dominate the infiltrative immune cells, which comprise up to 30% ~ 50% of glioma constituents (23, 24). It is initially conceived that TAMs shoulder tumor clearance through pro-inflammatory cytokine release and phagocytosis, which is validated by a recent study that uses activated TAMs for tumor containment (25). However, TAMs are one of the numerous culprits to be blamed in the immunosuppressive TIME glioma, whose reasons may lie in its components (26). The bone-marrow-derived macrophages (BMDMs) appear to be defined as CD11b^+-^ CD45^+^ CD49d^+^ macrophage population and recruited from peripheral circulating monocytes by glioma, whereas the tissue-resident CD11b^+^ CD45^-^ CD49d^-^ microglia (MG) are derived from erythro-myeloid progenitors (EMPs) in embryonic yolk sac without postnatal replenishment from peripheral mononuclear hematopoiesis (5, 8, 27). Of distinct ontogenies, infiltrating TAMs may lead to varying outcomes in patients (28). The gene signature of BMDM, rather than MG, is observed to be negatively associated with survival in LGG (29). To better comprehend the complex mechanisms, further studies are conducted concerning the interplay between TAMs and glioma.

Glioma attracts TAMs, especially BMDMs, by secreting chemoattractants, including C-C motif chemokine ligand 2 (CCL2), C-C motif chemokine ligand 3 (CCL3), C-X-C motif chemokine ligand 12 (CXCL12), C-X3-C motif chemokine ligand 1(CX3CL1), colony-stimulating factor 1 (CSF-1), and granulocyte-macrophage colony-stimulating factor (GM-CSF) (30–33). Typically, CCL2 and CX3CL1 are regarded as the two of the most important chemokines in directing BMDMs and MG migration, respectively. A study reports that low-grade glioma stem-like cells (GSCs) harboring *BRAF* kinase gene mutations (*KIAA1549:BRAF* fusion) express Ccl2 for circulating monocytes recruitment (34). In parallel, MG highly expressing Cx3cr1 are led towards glioma by Cx3cl1, which is secreted by glioma cells with NF1 mutation (35). Moreover, intercellular adhesion molecule 1 (ICAM1) silencing in the *IDH1* wild-type glioma cells is demonstrated to increase macrophage infiltration and potentially enhance anti-tumor functions like phagocytosis. There are alternative ways for TAMs to be recruited, of which the composition could be chemokine-dependent (8). For example, lysyl oxidase (LOX) expression is activated by yes1 associated transcriptional regulator (YAP1) in a PTEN-deficient GBM model to recruit macrophages, which in turn supports the GBM with Secreted Phosphoprotein 1 (SPP1) (36).

Heavily dependent on the specific environmental signals, such as interferon-γ (IFN-γ), tumor necrosis factor (TNF), and IL-4, macrophages usually polarize but are not confined to binary phenotypes, antitumor M1 and protumor M2 subtypes (37). CD68^+^ and CD163^+^ M2 polarized cells are more prevalent in the TIME, which is in line with their immunosuppressive properties (38). However, there are difficulties in applying the predefined dual classification to TAMs. The expression profiles of TAMs in the GL261 glioma model and RCAS transgenic system, known as murine glioma models, resemble other specialized macrophage subgroups rather than matching with either the M1 or the M2 polarity (39). Consistently, TAMs isolated from the patients’ biopsies co-express M1 and M2 genes frequently (29, 40). It may give rise to a novel classification suitable for TAMs, but the current paradigm distinguishing M1/M2 lays a solid basis for future refinement, which is CD40, CD74, CD80, CD86, MHC-II, and phosphorylated Signal Transducer and Activator Of Transcription 1 (STAT1) for the M1-like, while it is CD163, CD204, CD206, arginase-1(ARG-1), and phosphorylated STAT3 and STAT6 for the M2-like (30, 41).

Not only do the genetic alterations in TAMs result in new lineages, but they also involve glioma progression and lead to immunosuppression in the glioma microenvironment. The finding that the mouse GL261 glioma cells inoculated in *Tlr2* knockout mice grow a smaller tumor leading to longer survival of the host compared to the control indicates that the TAMs promote glioma progression *via* TLR2 signaling (42). TLR2 upregulates membrane type 1 matrix metalloprotease (MT1-MMP) and metallopeptidase 14 (MMP14) in MG to activate glioma-sourced MMP2 when responding to TLR2 endogenous ligands and thereby facilitate glioma invasion (43). TLR2 also increases matrix metalloproteinase 9 (MMP9) production, resulting in extracellular matrix (ECM) degradation (44). Of note, platelet-derived growth factor receptor (PDGFR) in glioma cells is induced by MG exclusively, which also promotes glioma progression (45). As for immunosuppression, TAMs produce CCL2 for recruiting CCR2^+^ MDSCs and CCR4^+^ Tregs (46).

Apart from the genetic influences, the distinct distribution of TAM components in glioma could be listed as a major factor contributing to immunosuppression as well. Recent single-cell RNA sequencing analyses reiterates that the BMDMs are more responsible for immunosuppression and unfavorable outcomes in glioma in contrast to the MG that mostly resides in the tumor periphery (29, 47–49). It also indicates that IDH mutation may correlate with TAM infiltration when the critical shift in the proportion of MG to BMDM between IDH mutation and IDH wild type is observed (49). Consistent with previous findings, it is ascribed to the BMDMs in the glioma center rather than the brain-resident MG occupying the peritumor area in terms of inhibited immunity in glioma (50). It leaves MG a dearth of credits since most effects are pinned on migratory macrophages. Indeed, the resident MG is capable of phagocytosis when meeting with glioma cells in response to myeloid checkpoint CD47- Signal regulatory proteinα (SIRPα) blockade *in vivo*. The MG even shows a dampened inflammatory response, making specific reeducation of the MG a promising strategy in glioma management (51). These reports indicate that specific and distinct roles of the TAM compartments are emerging with more in-depth investigations ongoing at single-cell resolution.

Moreover, studies reveal the crucial role of TAMs in glioma tumorigenesis, stemness, angiogenesis, invasion, and migration (52–55). Interleukin 1β (IL-1β) released by TAMs reprograms cellular metabolism by boosting glycolysis of glioma through the IL-1β-protein kinase-delta (PKCδ)-glycolytic enzyme glycerol-3-phosphate dehydrogenase (GPD2) axis, which promotes tumor proliferation and tumorigenesis (56). Specifically, it is C-C motif chemokine ligand 8 (CCL8) highly expressed by TAMs that promotes stem-like traits of GBM cells *via* the activation of ERK1/2 (54). Glioma angiogenesis is induced *via* vascular endothelial growth factor (VEGF) secretion (57). The fact that C-C motif chemokine ligand 5 (CCL5) modulates invasive and migratory behaviors of glioma through the phosphorylation of calmodulin-dependent protein kinase II (CaMKII) renders CCL5 and CaMKII interesting targets to halt glioma progression (55). More related reports are needed for an improved understanding of how TAMs interact with glioma and modulate the TIME so that therapies targeting TAMs would be boosted.

### Myeloid-Derived Suppressive Cells

As a critical part of the suppressive network, myeloid-derived suppressive cells usually accumulate under pathologic conditions (58). The enrichment of MDSCs predicts glioma malignancy, poor prognosis, and low responses to treatments (59). Interestingly, the Bruyère group finds that the inhibition of chemokine C-X-C motif chemokine ligand 2 (CXCL2) expression in Hs683 glioma cells results in impaired cell proliferation (60). There is a related report showing that it is the CXCL2-CXCR2 axis that mediates MDSCs recruitment in the tumor (61). Targeting CCR2 with CCX872 not only reduces infiltrated MDSCs but also augments immunotherapy efficacy (62). Still, more mechanism studies uncovering how MDSCs are recruited to glioma are encouraged.

MDSCs mainly consist of CD14^+^ CD15^-^ monocytic MDSCs (M-MDSCs) and CD14^-^ CD15^+^ granulocytic or polymorphonuclear MDSCs (PMN-MDSCs). Some evidence shows that PMN-MDSCs predominates the blood and M-MDSCs mainly distribute throughout glioma tissue, while others supported the opposite, which may require more studies to corroborate (63–65). The two subsets of MDSCs could be differentiated from each other based on genomic entities, biochemical markers, and biological functions, especially the capacity to inhibit immune responses. Interestingly, hypoxia-stimulated glioma-derived exosomes containing microRNAs are reportedly associated with MDSC proliferation, differentiation, and activation (66, 67).

MDSCs suppress immunological reactivities in a variety of ways. They cripple TAMs and DCs for antigen presentation impairment, inhibit anti-tumor responses by NK cells and cytotoxic T cells, and induce inhibitory regulatory T cells (Tregs) (68–71). MDSCs modulate TAMs, the most common immune cells in glioma, to attain the suppressive goal. Through crosstalk with MDSCs releasing IL-10, macrophages are skewed towards the M2 phenotype producing less IL-12 and more IL-10 (72). Similarly, it claims that MDSCs also disturb DC-mediated T cell stimulation by IL-10 (73). MDSCs additionally inhibited NK cells with ROS, TGF-β1, and NKp30 (74–76). The main strategy that MDSCs employed to interfere with T cells is through signal transducer and activator of transcription 3 (STAT3)-induced ROS and reactive nitrogen species (RNS) as oxidative stress, which leads to an increased level of arginase 1 (ARG1) and inducible nitric oxide synthase (iNOS) and thereafter L-arginine depletion, cell cycle arrest, and even apoptosis (70, 77, 78). The oxidative stress additionally renders T cells anergic and suppressive by reducing CD3 ζ chain expression and inducing nitrosylation of the IL-2 pathway (78). It also inhibited T cell migration when CCL2 is under nitration (70).

Beyond modulation of immune cells, some molecules, like nitric oxide (NO), prostaglandin E2 (PGE2), and programmed cell death 1 ligand 1 (PD-L1), are also associated with MDSC-mediated immunosuppression (79–82). The suppressive function of regulatory B cells (Bregs) is augmented by PD-L1 derived from MDSCs and thereby impeding CD8^+^ T cell activation (82). Moreover, tumor-associated macrophages (TAM) can be renewed by M-MDSCs or monocytes differentiation, which is facilitated by HIF-1α along with CD45 tyrosine phosphate and STAT3 regulation in hypoxia (83–85).

### Neutrophils

As the first-line sentinel of host defense for tissue homeostasis, neutrophils rapidly migrate to the tumor site in response to many signals like IL-8 and IL-1β (86). Glioma-derived IL-8 recruits neutrophils to infiltrate, while the recruited ones produce a magnitude of neutrophil extracellular traps (NETs) stimulating the NF-κB signaling pathway in GBM cells and promote IL-8 secretion *via* HMGB1 binding to the receptor for advanced glycation end products (RAGE) (87). Glioma progression accompanying neutrophil recruitment is also mediated by the long non-coding RNA LINC01116-triggered IL-1β upregulation (88). It is observed that both circulating and infiltrative neutrophils increase with glioma pathological grade, which predicts poor prognosis for patients (78). Besides, a recent study proposes that ferroptosis could be the nature of “necrosis” typically identified in GBM, which is mediated by the infiltrating neutrophils and the myeloperoxidase-containing granules (89). In contrast to their proinflammatory role, activated neutrophils (or granulocytic MDSCs) characterized by high plasma levels of IL12p70 promote glioma malignancy (90). Elastases secreted by infiltrative neutrophils nearby also accelerate the infiltration in glioma (91).

When treating glioblastoma with anti-VEGF therapy, increased neutrophil infiltration advances glioma mesenchymal transition and promotes proliferation of GSCs through upregulation of S100A4 (92). Targeting S100A4 can also sensitize glioma cells to bevacizumab treatment. The protumor role of neutrophils is further strengthened since depleting glioma-associated neutrophils with a monoclonal antibody against Ly6G^+^ neutrophils prolongs survival in a preclinical GBM murine model (93). Neutrophils are also involved in the resistance to PD-1 inhibitors, which is revealed by the improved therapeutic efficacy of combinational treatment with antineutrophil and PD-1 inhibitors (94). However, more in-depth studies concerning the molecular mechanism mediated by neutrophils would provide better comprehension for the development of glioma therapy resistance.

Yet the detailed mechanism for neutrophil recruitment to glioma remains largely elusive. How the interaction between neutrophils and glioma in the local immune microenvironment works still needs to be intensively investigated. Since tumor-associated neutrophils generally exhibit functional plasticity and polarization, represented by anti-tumor N1-like and pro-tumor N2-like states, it would inspire more treatment strategies if the stimuli for neutrophil reprogramming and differentiation were uncovered.

### Dendritic Cells

Due to the overestimation of MG as major antigen-presenting cells in CNS, DCs have not been gaining sufficient attention until recently (95, 96). DCs serve as indispensable sentinels of adaptive immune responses through internalizing surrounding antigens for presentation (97, 98). DCs are well-known for their activation of NK cells, T cells, and Tregs. One majority subtype of DCs is mostly CD11c^+^ myeloid conventional dendritic cells (cDCs), another group is CD11c^-^ plasmacytoid dendritic cells (pDCs) (99, 100). Relative to cDCs aiding CD4^+^ Th1 differentiation and CD8^+^ T cell activation, pDCs are more of interest because of their secretion of IFN-α, which directly stimulates antitumor immunity (100, 101).

Nonetheless, there are some studies casting doubts on immunogenicity generated by pDCs. The compromised elaboration of IFN-α, impaired antigen presentation, and increased Treg infiltration are witnessed in a murine glioma model (96). As restoration, the survival of the tumor-bearing mice extends and infiltrative Treg cells diminish when pDCs are selectively excluded. A similar report also argues that pDCs are at play in promoting tumor progression and immunosuppression under the influence of granulocyte-macrophage colony-stimulating factor (GM-CSF) (102). DCs are induced to produce IL-10 and thereby inhibit T cell *via* its poliovirus receptor (PVR) -immunoreceptor tyrosine-based inhibitory motif (ITIM) interaction with T cells (103). Further, immature DCs characterized by low expression of CD80 and CD86 contribute to tolerance in T cells (104).

### Mast Cells

The presence of mast cell (MC) in mouse and human glioma mirrors the entanglement of inflammation and cancer (105). It is demonstrated that the endogenous stem cell factor (SCF) largely contributes not only to the expansion of the glioma-associated MCs but also to the localization of the MCs in the vicinity of the tumor blood vessel and glioma cells along with the CXCL12/CXCR4 axis (105). Glioma-derived macrophage migration inhibitory factor (MIF) also recruits MCs to glioma through signal transducer and activator of transcription 5 (STAT5) signaling in a malignancy-dependent manner (106). Interactively, recruited MCs release a variety of mediators to inhibit glioma progression and induce tumor differentiation by downregulating GSK3β expression (107).

### T Cells and Regulatory T Cells

Standing in as infiltrative T cells are CD4^+^ helper T cells (Th), CD8^+^ cytotoxic T cells, and CD4^+^/CD25^+^/FoxP3^+^ Tregs which lead an exhausting life in the suppressive microenvironment of glioma (9). Although constantly releasing quantities of proinflammatory IFN-γ for T cell recruitment, CD4^+^ T cells upregulate the expression of inhibitory co-receptors like programmed cell death protein 1 (PD-1), cytotoxic T lymphocyte-associated antigen (CTLA), lymphocyte-activation-gene-3 (LAG-3), and T cell immunoglobulin domain and mucin domain-3 (TIM-3) (108). CD8^+^ T cells remained largely inactive, being in line with their counterparts. The inactivation of T cells may also come from IL-10 and TGF-β released by glioma cells (9). In addition, attracted by mediators such as CCL2 and IDO, Tregs contribute greatly to immunosuppression (108, 109).

Increased T cell infiltration is related to favorable outcomes in glioblastoma patients (110). However, only limited immune response mediated by T cells is permitted in CNS, especially in the glioma microenvironment. The maturation of T cells is inhibited by tumor-derived Fas ligand (111). The activation of T cells is also inhibited by the IDO and TGF-β released from MG. Lack of co-stimulatory CD40, CD80, and CD86 expression on TAMs and glioma cell surface disengage T cell binding (112). T cell-mediated immunity in glioma is further drained through apoptosis induced by PD-1 and CTLA (9). An additional observation shows that GCN2 kinase results in T cell anergy and lack of proliferation in response to tryptophan depletion by IDO (113). Another GCN2-focused report highlights the importance of GCN2 as an amino acid sensor preventing CD8+ T cell apoptosis under amino acid stress in a murine glioma model (114).

Like two sides of the same coin, regulatory T cells ordinarily orchestrate balance and keep hyperactive immunity, including overactivated T cells, in control. In contrast to the T cells, infiltrative Tregs increase with glioma grade, predicts poor survival, and relates to recurrence (115, 116). It recruits Treg cells towards glioma using attractants such as CCL2 and IDO (46). The IDO expression not only involves Treg recruitment and immunosuppression thereafter but also plays a critical role in the Treg cell expansion by interacting with mTOR (117). Although it seems obvious and solid to conclude the interplay between the Treg and glioma, it is of great importance to dig deeper into the encouraging topic.

Furthermore, largely dependent on Tregs for either pro- or anti-tumor polarization are T helper 17 (Th17) cells (118). Tregs may induce Th17 polarization towards IL-10 producing cells rather than IFN-γ secretion in the context of TGF-β *in vitro* (118). Another Th17 study consistently reports that the TGF-β1 stimulated Th17 cells may lead to the permissive TIME in glioma by releasing IL-10 (119). The confirmation of Th17 cell infiltration in the glioma tissues gives rise to the investigation of the role of IL-17, one of the major mediators and hallmarks of the Th17 cells, which promotes glioma proliferation and migration through the activation of PI3K/Akt1/NF-κB-p65 axis (120).

### Natural Killer Cells

NK cells detect and precisely execute cancer cells. They act under the balance between activating and inhibitory signals once approaching the susceptible (75). Given the minor proportion of infiltrative CD45^+^ cells, NK cell activity is blunted close to non-functional. Glioma cells utilize MHC-I molecules binding with the inhibitory killer cell immunoglobulin-like receptor (KIR) to evade termination (121). As previously mentioned, NK cells are also inhibited by MDSCs *via* NKp30/NRC3 or NKG2D, and ensuing reduced IFN-γ production (75).

### Cancer-Associated Fibroblasts

Given the fact that fibroblasts are associated with the progression and metastasis of many malignancies, it is reasonable to postulate that the cancer-associated fibroblasts (CAF) in the tumor niche of glioma may contribute to the proliferation or invasion of glioma (122–124). However, pieces of evidence related to fibroblast entities and existence in the brain remain scarce. A study claims that tumor-associated mesenchymal stem-like cells (tMSLCs), presumably as reminiscent of fibroblasts in other tumors, correlate with the poor prognosis of the GBM and enhance the invasiveness of GBM by force-mediated ECM remodeling through CCL2/JAK1/MLC2 signaling (125). And this pro-invasive effect brought by CAFs in glioma could also be mediated by the secretion of CXCL14 (126). Furthermore, it reveals that long non-coding RNA (lncRNA) HOXA transcript antisense RNA, myeloid-specific 1 (HOTAIRM1), is upregulated in the malignantly transformed fibroblasts derived from an orthotopic model and regulates TGF-β *via* miR-133b-3p to promote malignancy (127).

### Cytokines, Chemokines, and Extracellular Molecules

Elevated levels of inhibitory cytokines, such as IL-10 and TGF-β, overthrow the balance with proinflammatory molecules contributing to the immunosuppressive microenvironment around glioma. It is interesting that IDO produced by glioma cells or pDC activates Treg cells and impedes T cell activity through tryptophan depletion (109). Besides, kynurenine, a metabolite of tryptophan, induces T cell apoptosis and polarizes Tregs by upregulating Foxp3 expression (128, 129). However, preclinical trials targeting IDO hold less promise than is expected (130).

Moreover, interference with *LGALS1* expression, which encoded Galectin-1 in the immune system, reduces MDSCs infiltration and immunosuppressive cytokine secretion (131). LAG3 binds to Galectin-3 (Gal-3) and MHC-II for CD8^+^ T cell inhibition and inhibitory signal transmission, respectively (132, 133). It is reported that LAG-3 overexpression depletes CD8+ T cells (134). By binding to Galectin-9 (Gal-9), TIM-3 regulates T cell depletion and contributes to immunosuppression and even immune evasion. Limited arginine resulted from high levels of infiltrative myeloid cells-derived arginase also restrains immunocytes’ survival.

## Autophagy Modifies the Interplay Between Glioma and the Tumor Immune Microenvironment

**Figure 2:**
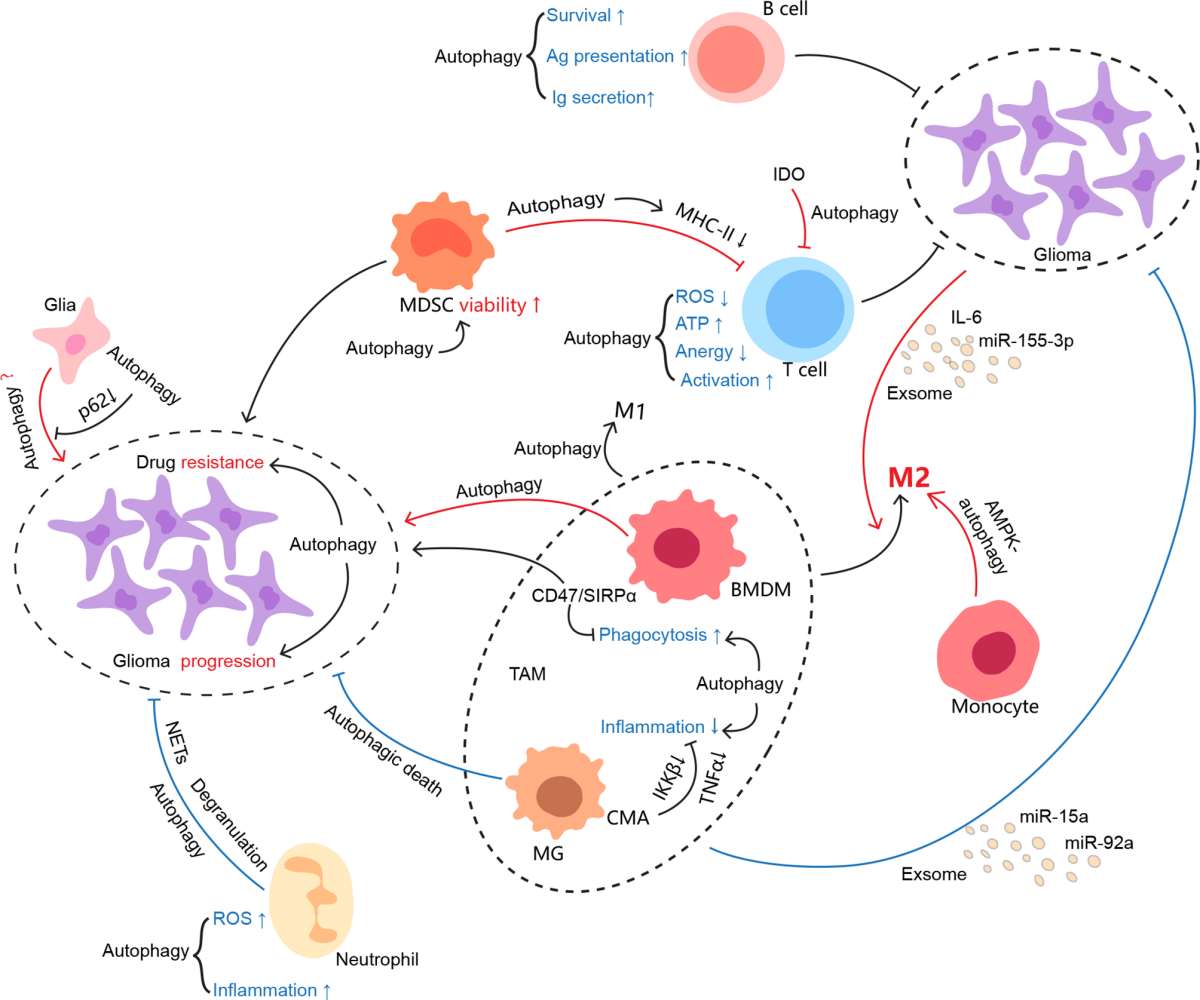
Autophagy involvement in the regulation of the glioma immune microenvironment. As one of the evolutionarily conserved processes, autophagy keeps cellular homeostasis not only for immunocytes but also for tumor cells. It may be manipulated by conflicting sides in favor of their profits. However, it seems that autophagy somehow is not capable of either ensuring the physiological function of immune cells or restraining glioma growth in the glioma immune microenvironment. The interconnections between the cells in the TIME are colored if autophagy is involved, where red indicates protumor effects and blue indicates antitumor effects.

Autophagy becomes an interesting element in glioma due to paradoxical roles in glioma oncogenesis, progression, metastasis, and therapy resistance. Whether autophagy favors or hinders tumors may highly depend on stimuli, cell type, and specific stage of tumor cells. As a physiological part working at a base level, autophagy degrades misfolded proteins and recycles organelles to reduce unsolicited ROS production, protein aggregates, and further damages DNA especially under stress (135). Ironically, it may apply the used tricks for normal survival to nurture tumor growth once the cells trend towards malignancy (136, 137). Herein, autophagy in glioma cells per se suits the case, as well as each component of its surrounding immune microenvironment, and thereby tweaking the interplay between glioma and the TIME (**Figure 2**).

### Autophagy in Glioma Cells

As one hypothesizes an “inhibition-loss-promotion” model in an attempt to unify the conflicting roles of autophagy in cancer development, the model may apply to glioma with some revisions (12). Proficient autophagy may counteract the accumulated genetic defects when glioma cells are undergoing malignant transformation. Autophagy exhibits suppression on oncogenesis by degrading p62, so that mitochondrial damages, ROS-mediated oxidized molecules, and unstable genome mutations are averted (138, 139). Given that autophagy degrades carbohydrates, proteins, and lipids into sugars, amino acids, and fatty acids, respectively, to fuel cellular metabolism maintaining homeostasis, it still displays bipolar effects in glioma initiation after transitory “loss” or compromises at a very early phase of malignant transformation from healthy cells (140). Once glioma is established, autophagy responds more variably to cellular and environmental stimuli than expected with glioma growing and progressing.

In a KRAS-driven GBM mouse model, autophagy inhibited by downregulating *Ulk1*, *Atg7*, and *Atg13* disrupts tumor growth and development. The capacity of KRAS-expressing glial cells for colony formation and survival in low-serum conditions is inhibited when autophagy is inhibited (141). It indicates that autophagy is vital to facilitate oncogenic transformation. The malignant, heterogeneous, and recurrent properties of GBM may source from GSCs, whose pluripotency and proliferation highly implicate autophagy (142–145). For instance, autophagy is manifested to degrade Notch1 and thereby modulate GSC tumorigenicity (146). The GSC tumorigenicity is also restrained by the overexpression of microRNA-93 (miR-93) targeting autophagic regulators, including Beclin-1, ATG5, ATG4B, and p62 (147).

Furthermore, GSCs activate autophagy through Bcl-2 nineteen-kilodalton interacting protein 3 (BNIP3) to habituate themselves to hypoxia (148, 149). More than adapting to hypoxia, BNIP-3-mediated autophagy facilitates GBMs thriving and contributes to resistance to chemotherapies *via* the MT1-MMP-JAK-STAT axis (150). As confirmation of autophagy modifying GSC survival, autophagy inhibitor quinacrine sensitizes GSCs to TMZ by triggering ferroptosis (146). However, autophagy induction *via* mTOR inhibition in GSCs promotes differentiation and disrupted autophagy increases stemness, which suggests that manipulation of autophagy influences GSCs formation and further GBM malignancy (151–153).

Emerging evidence shows that autophagy is implicated in glioma progression. Autophagy-related protein LC3 and p62 expression levels are negatively associated with glioma prognosis, especially with high-grade glioma prognosis, suggesting potential links between autophagy and glioma progression (154). Knockdown of autophagy-related 4C cysteine peptidase (ATG4C) repressed glioma progression by arresting tumor cells at the G1 phase and promoting apoptosis (155). Overexpression of maternal expression gene 3 (Meg3) encoding a critical non-coding RNA promotes epithelial to mesenchymal transition, migration, invasion, and autophagy in glioma. The employment of autophagy inhibitor CQ partially hampers glioma progression, suggesting autophagy involvement (156). Silencing of lysosome enzyme alpha-l-fucosidase 1 (FUCA1) impedes U87 and U251 glioma cell proliferation, whereas the growth inhibition appears less significant when the cells are co-incubating with 3-MA (157). It implies that downregulation of FUCA1 may lead to autophagy-associated cell death, considering increased LC3-II/LC3-I ratio, Beclin-1, and ATG-12 expression level. Interestingly, antidepressant imipramine augmented with P2Y12 inhibitor ticlopidine elicits autophagy-associated cell death, which limits glioma cell growth *in vitro*, hinders tumor progression *in vivo*, and prolongs survival of glioma-bearing mice (158). Together, autophagy closely involves glioma progression, while its reputation mostly lies in therapy resistance.

The point that autophagy mainly contributes to glioma resistance to therapies prevails and is corroborated by a majority of studies. TMZ stands as a clinical routine for GBM treatment because of its high efficacy, BBB permeation, and tolerable adverse effects (16, 159). As suggested, cytoprotective autophagy saves cells from stresses, including drug, radiation, and hypoxia cytotoxicity from death (160–162). In the case of drug resistance, for instance, GSC−derived PD−L1−containing exosomes activate AMPK/ULK1-mediated autophagy to increase TMZ−resistance in GBM (163). And ATG4C depletion impairs TMZ-resistance and improves the susceptibility of glioma to TMZ (155). Similarly, autophagy is transiently induced by acute TMZ treatment *via* transitory activation of the AMPK-ULK1 axis, while CQ blocks the autophagy and enhances the efficacy of TMZ in glioma cells (18, 164). CQ and its analogs have therefore been an alternative adjuvant to potentiate the TMZ treatment. Moreover, T-LAK cell-originated protein kinase (TOPK) inhibits autophagy by phosphorylating ULK1 in glioma cells and promotes glioma resistance to TMZ (165). TMZ-stimulated autophagy is also inhibited by the lncRNA DLEU1 knockdown, which increases glioma susceptibility to the drug cytotoxicity at the same time (166). In addition, lncRNA CASC2 sponges miR-193a-5p to reduce autophagy and potentiate TMZ efficacy *via* mTOR upregulation (167). LncRNA AC023115.3 induced by chemotherapy, in turn, improves chemosensitivity of glioma to cisplatin by competing with miR-26a, thereby releasing GSK3β to inhibit autophagy (168).

The role of autophagy in glioma radiation therapy is currently controversial. Autophagy is triggered to shelter glioma cells from death due to the toxicity of free radicals, misfolded proteins, and damaged organelles mediated by radiotherapy (169–171). It is demonstrated that radiation augments autophagic flux by upregulating mammalian sterile-20-like kinase 4 (MST4), which phosphorylates and motivates ATG4B in GBM, whereas inhibition of autophagy *via* ATG4B blockade improves sensitivity to radiation (170). Targeting Beclin-1-mediated autophagy with miR-17-5p expression also improves the radiosensitivity of glioma (172). However, radiation-induced autophagy may also promote apoptosis in glioma (173). There could be an explanation for the conflicting results that glioma cells respond variably to radiation simply out of distinct individual sensitivity, but the intensity and duration of radiation should also be considered (174).

A large body of literature regarding autophagy-mediated therapy resistance predisposes autophagy to be cytoprotective, but the fact that overactivated or insufficient autophagy under drug perturbation drives glioma cells to death should not be neglected. It is demonstrated that autophagy-associated cell death after TMZ or ionizing radiation treatment is relieved by downregulation of Beclin-1 or ATG7 with siRNA (175). In light of the extent of triggering autophagy-associated death, coordinated autophagy modulation with autophagy inducers followed by inhibitors and TMZ treatment may overcome chemotherapeutic resistance in GBM. It is noticeable that mitochondrial respiration and oxidative phosphorylation is greatly interfered with by the disrupted autophagic system under autophagy modulation (176). Though more clinical translation is in need, tweaks in the autophagic system using autophagy inducers and consecutive inhibitors would doubtlessly galvanize autophagic intervention for chemoresistance.

How autophagy shuttles between cytoprotective and cytotoxic roles in glioma, especially in terms of therapeutic resistance, probably depends on doses and duration of treatment to decide a compatible pathway (16). It indicates that autophagy may act as a survival strategy under relatively mild therapies, such as low dose and short term, but consistent treatment with maximum dosage could induce autophagy-associated cell death (164). Of all the possible mediators for protective autophagy initiation, surging adenosine triphosphate (ATP) is observed to have a key role in promoting autophagy for drug toxicity adaption, especially in glioma cells on TMZ administration (177). Disruption of energy balance with drug targeting mitochondria activity is demonstrated to induce AMPK phosphorylation and inactivate mTORC1 and thereby suppress aggressive properties of glioma cells (178). Since TMZ in co-treatment with autophagy inhibitor CQ induces ROS production and augments cytotoxicity, it is also reasonable to hypothesize that ROS level may drive the shift of autophagy from cytoprotection to cytotoxicity with the additional consideration that hydrogen peroxide accumulation leads to apoptosis or necrosis (179, 180).

Although the hypothetical theory seems to coordinate autophagy’s conflicting roles in therapy resistance, autophagy in glioma development remains misunderstood. How to determine any mediators or specific markers for autophagy altering from the beneficial to the lethal is yet to be answered in glioma progression. It may display context-dependency, such as the ROS and ATP level in the intra- or intercellular microenvironment (181). Or, it is reasonable to speculate that mild stimuli give rise to cytoprotective autophagy, whereas harsher conditions lead to autophagy-associated death.

### Autophagy in TAMs

It remains controversial to assess the contribution of autophagy in the components of the glioma-related immune microenvironment. Based on the currently incomplete understanding, autophagy mainly involves TAM generation, function, and polarization (182, 183). Autophagy induced by c-Jun N-terminal Kinase (JNK) activation and disruption of ATG5 cleavage promotes monocytes differentiation into macrophages, produces cytokines, and prevents monocyte apoptosis (184). For phagocytosis of debris, macrophages degrade phagocytosed cells *via* LC3-associated phagocytosis, of which blockade would improve anti-tumor immunity (185). Interestingly, disrupting the CD47-SIRPα axis (“don’t eat me” signal) with specific fusion protein binding augments phagocytosis of macrophages, triggers cytoprotective autophagic flux in glioma cells, and further improves not only macrophage but also CD8+ T cell infiltration if combined with autophagy inhibitors (186, 187).

Impairment of the autophagy-mediated phagocytosis in MG also stimulates inflammation (188). A study shows that Beclin-1-mediated autophagy may regulate neuroinflammation *via* NRLP3 degradation in murine MG (189). On top of that, rapid loss of IKKβ protein and TNF-α is mediated by CMA in microglia, indicating the critical role of CMA in controlling inflammation (190). These discussed studies suggest that impaired autophagy in TAMs may enhance inflammation, but it should be reconsidered for the simultaneous inhibition of phagocytosis.

In an immunosuppressive microenvironment, TAMs incline to polarize toward the M2 phenotype facilitating tumor progression and metastasis. Interestingly, autophagy contributes greatly to the M2 polarization, namely *via* the STAT3 pathway (191–193). Several studies modify the TAM state into a specific activation state by targeting autophagy (194–196). Monocytes respond to colony-stimulating factor-1 (CSF-1) through AMPK-mediated autophagy activation and differentiate into M2 macrophages (188, 197). Nonetheless, there are always exceptions to predisposing TAM to the M2 phenotype, which presumably depends on tumor types and local milieu. For instance, it is reported that inhibition of mTOR, a key switch of the autophagy pathway, skews TAM polarization to the M1 phenotype, resulting in increased IL-12, decreased IL-10, and reduced tumor angiogenesis (195). Collectively, it may hold promise in targeting autophagy to modulate TAM polarization and function, but more investigations are required.

### Autophagy in MDSCs and Neutrophils

MDSCs contribute greatly to the suppressive environment around glioma and contrive autophagy to maintain themselves under inhospitable conditions. It is understood that autophagy promotes MDSC viability. Either neutralization of high mobility group box protein-1 (HMGB1), a damage-associated molecular pattern, induces autophagy, or inhibiting autophagy directly increases apoptosis in MDSCs. Furthermore, interference with autophagy in MDSCs hampers tumor growth by endorsing antitumor responses. MDSCs enhance MHC II expression due to compromised autophagy and reduced lysosomal degradation so that tumor-specific CD4^+^ T cells are efficiently activated (198).

Neutrophils exist and survive in the suppressed environment of glioma although the mechanisms of neutrophil recruitment to glioma are poorly defined (199). The specific role that autophagy plays in the context of glioma is worthy of reevaluation, even if the implication of autophagy in neutrophil granulopoiesis, phagocytosis, degranulation, and neutrophil extracellular trap (NET) formation is well-documented (200). In particular, neutrophils also require autophagy as a key regulator for building NADPH-oxidase-mediated reactive oxygen species (ROS) and inflammatory activity (201).

### Autophagy in Tumor-Infiltrating Lymphocytes

Autophagy generally maintains cellular homeostasis and relieves lymphocytes from accumulating stress (202, 203). Notably, reports are claiming that autophagy-mediated metabolism in T cells modulates T cell activity (204, 205). That T cells heavily rely on autophagy for ATP supply is not limited to survival guarantee, but is also essential when T cell receptor (TCR) engagement and CD4^+^ The cell activation cause an increased demand for energy (205). Following activation of CD4^+^ Th cell, common γ-chain cytokines represented by IL-2 and IL-4 also mediate regulation of autophagy in Th cell with JAK signaling involvement (206). It indicates that induction of autophagy in CD4^+^ T cells in response to TCR activation secures the T cell engaging in effector responses accordingly, and exempts itself from the energy otherwise (207). Upon TCR stimulation, however, the planned entry to the S phase is unexpectedly perturbed thanks to accumulated cyclin-dependent kinase inhibitor CDKN1B in autophagy-deficient T cell, suggesting a key role of autophagy in T cell proliferation (208).

Autophagy fuels CD4^+^ T cells and it meets the energy demands of other T cell populations as well. It is dynamically regulated throughout CD8^+^ T cell activities, including proliferation, memory generation, function, and survival (209). In particular, CD8^+^ T cells depleted of *Atg5* or *Atg14* are metabolically reprogrammed to glycolytic consumption and display an enhanced effector memory cell activity against the tumor (210, 211). The report implies that the shift towards glycolytic metabolism instead of autophagy in *Atg5* ^-/-^ T cells leaves less carbon available for methylation and results in epigenetic alterations.

Not does autophagy only adjust to activity or environmental cues but also to distinct T cell lineages (212). Tregs depleted of *Atg5* or *Atg7* show upregulation of mTOR complex 1(mTORC1) and glycolysis, defective functional integrity, and increased apoptosis (213). Interestingly, it rescued the Foxp3 instability phenotype in autophagy-deficient Tregs by inhibiting pyruvate dehydrogenase kinase and glycolysis.

Overall, those studies imply that autophagy fulfilling the energy needs of lymphocytes is subject to specific environmental context, or exact activity, or cell type in a variety of pathways (212). It is plausible to hypothesize that autophagy in those infiltrating T cells is occluded, at least partially, or are diverted to degrade anti-tumor molecules only, or other undiscovered mechanisms contribute to the “non-functional” T cells in the glioma immune microenvironment. Therefore, studies at a single-cell resolution are required for a complete understanding of autophagy in shaping the protumor microenvironment.

## Redefine the Interconnection Between Glioma and the Immune Microenvironment From an Autophagic Perspective

Autophagy is generally deployed by multiple types of cells in glioma not only for energy gratification and integrity maintenance, but also for essential functions, such as cytokine production, phagocytosis, and antigen presentation (182). Therefore, the interconnections between glioma and the TIME would be impacted if the energy supply or the cellular context of these cells alters with malignancy progressing. Substantial bioinformatic analyses raise awareness of the autophagy-associated links between glioma and the components of TIME predicting a wide area of intensive basic research (214–219). It has been anticipated by distinct autophagy gene signature-based prognostic risk models that there is more immune cells infiltration in the sets with higher autophagy risk scores, implying latent autophagy-involved associations between autophagy and immunity in glioma (214, 218, 220). However, interpretation for the “coincidental” intertwinement between autophagy and immune responses may require future experimental evidence. It is also worthy of investigation to determine whether it is a cause-effect relation or coincidence and to identify specific cell populations that manipulate autophagy and thereby renovate the immune microenvironment in glioma.

Hypoxia stimulates glioma cells to release exosomes into the peritumor area for intercellular communication. Since TAMs dominates the infiltrating immunocytes and autophagy is highly involved in M2-like macrophage, a piece of evidence illustrates that the glioma-derived exosomes mainly containing IL-6 and miR-155-3p initiate autophagic activities in TAMs and promote M2-like polarization *via* IL-6-pSTAT3-miR-155-3p-autophagy-pSTAT3 positive feedback loop (221, 222). It exposes the bridging role that autophagy plays in glioma-sourced immunosuppression. M2 macrophage-sourced exosomal miR-15a and miR-92a are reversely corroborated to inhibit glioma invasion and migration *via* phosphatidylinositol-3-kinase (PI3K)-AKT-mTOR pathway (223). On the other hand, LPS/IFN-γ-stimulated MG can trigger autophagy-dependent death in glioma cells that are resistant to death ligands, like TNF α and TRAIL (224). Although the specific molecular elements restoring the anti-tumor function of MG are of utmost interest, the report exposes that MG-mediated autophagic death may overcome apoptosis resistance in glioma (225).

In addition to exosomal microRNA-mediated autophagic activities, the researchers shed light on the employment of classic autophagy modulators to improve the therapeutic efficacy of immune checkpoint inhibitors (41). According to the report, rapamycin treatment combined with hydroxychloroquine (HCQ) reduces M2-like polarized macrophages *in vitro* and augments both M1/M2 and CD8/CD4 ratio in the GL261 model. The phagocytic capacity of treated macrophages is also enhanced (41). Further, dropping the expression level of CD47 and SIRPα on glioma cells and macrophages resulted from the combination treatment suggests the deteriorating anti-phagocytic ability of glioma (41, 186). Other mechanisms influencing the TIME may include the evidence that glioblastoma promotes CMA and thereby wearing off the immunogenicity of pericytes, which is reversed by the CMA inhibition (226).

Besides, a novel nanodiamond carrying doxorubicin (Nano-DOX) is designed and corroborated to trigger autophagy instead of apoptosis in GBM. The Nano-DOX further stimulates GBM cells to emit antigens and damage-associated molecular patterns (DAMPs) leading to the boosted activation of DCs (227). Moreover, the liposomal honokiol and disulfiram/copper codelivery system (CDX-LIPO) is demonstrated to be a remarkable autophagy initiator in glioma cells and induces immunogenic cell death which results in enhanced activation of anti-tumor immunity. The CDX-LIPO is developed to inhibit the mTOR signaling pathway to promote autophagy, shift the M2 TAM towards the M1 type, and even interfere with glucose metabolism and lactate production. It also triggers immunogenic cell death, promoting the maturation of antigen-presenting cells and, further, the activation of T cells. The TIME in glioma is thus remodeled, marked by M2-polarized TAMs, matured DCs and NK cells, activated cytotoxic T cells, and repressed MDSCs, along with diminished glycolysis and lactate metabolism (228).

### Current Autophagy-Adjuvant and Autophagy-Related Therapies Against Glioma

As is shown in the extensive studies discussed previously, there are generally two directions to manipulate autophagy against glioma. One method of autophagy intervention is to overcome protective autophagy stimulated by drugs and repurpose the lethal condition to induce cell death. For instance, it has long been discussed that TMZ treatment at clinically available dosage induces autophagy in glioma cells for survival in adverse conditions (229). The TMZ-induced autophagy may require ERK1/2 signaling (230). The therapeutic efficacy of TMZ is further improved with later autophagy inhibitor bafilomycin or CQ administration. Recent studies unravel the mechanisms of TMZ-stimulated autophagy including, but not limited to, mitochondrial and endoplasmic stress, O^6^-methylguanine adducts generation, and ATP surge (177, 231, 232).

The addition of CQ or HCQ to the conventional glioma treatment TMZ represents a breakthrough. These quinolone derivatives initially used as antimalarial and rheumatological drugs are the only autophagy modulators that have been extensively researched and even tested in clinical trials for glioma combinational therapies (NCT02378532 and NCT00486603) (233, 234). Particularly, CQ in combination with TMZ renders U87, U251, and LN229 cells susceptible to TMZ by interfering with GRP78-dependent and PI3KC3-BECN1-dependent autophagy and cleaving poly ADP-ribose polymerase (PARP) (18). It is revealed that mitophagy is additionally inhibited by the CQ cotreatment with TMZ resulting in ROS accumulation (179). Other synergistic effects of combinations of CQ and TMZ may lie in the p53-mediated apoptosis induction or p53-independent cell cycle arrest in glioma cell lines (235). However, CQ- or HCQ-based conclusions merit further consideration due to their autophagy-independent off-target effects (137). Research focusing on more precisely targeted inhibitors of autophagy are highly welcomed for antitumor drug development.

However, a few clinical trials have been conducted to translate the promising treatment efficacy of CQ and TMZ into the application. A total of 30 postoperative GBM patients are recruited into a randomized, double-blinded, and placebo-controlled trial to evaluate the efficacy of CQ as adjuvant therapy for glioma (234). Despite the lack of statistical significance, the median survival of CQ-treated patients being 24 months is approximately two times longer than the control group being 11 months, which may be more definitive and more generalized if enlarging the sample size is permitted. A more recent phase I/II cohort focuses on the efficacy of HCQ along with concurrent radiotherapy and TMZ administration in newly diagnosed GBM (233). Nevertheless, the maximum tolerated dose for HCQ is 600 mg/d due to severe dose-limiting toxicity, at which autophagy may not be sufficiently and consistently inhibited in patients. As a result, any significant improvement in overall survival is hardly detected (233). The conflicting results between basic studies and clinical trials encourage the development of more tolerable, BBB-penetrable, and potent autophagy modulators. More novel options in conjunction with TMZ for glioma management are currently being explored and evaluated, such as combined TMZ/SAHA therapy (**Table 1**) (236).

**Table 1:** Autophagy-related therapy against glioma.

Autophagy-Related Therapy	Mechanism	Effects on Autophagy	Autophagy Type	Experiment Setting	Refs
TMZ	ROS induction	Activation	Protective autophagy	*In vitro*: U87 and U251	(177)
TMZ + CQ	PARP cleavage, apoptosis induction	Inhibition	Protective autophagy	*In vitro*: LN229 and U251 *In vivo*: U251	(18)
	ROS induction	Inhibition	Protective autophagy	In vitro: U87 and C6	(179)
	Induction of p53-dependent apoptosis and cell cycle arrest	Inhibition	Protective autophagy	*In vitro*: U87 and U373	(235)
	Induction of toxicity	Inhibition	Protective autophagy	Clinical trial Phase I	(234)
TMZ + HCQ	Induction of toxicity	Inhibition	Protective autophagy	Clinical trial Phase I/II	(233)
TMZ + SAHA + CQ	Induction of apoptosis, H3 and H4 histone acetylation	Inhibition	Protective autophagy	*In vitro*: U251	(236)
TMZ + Curcumin + CQ	Induction of DNA damage, inhibition of PI3K/AKT ERK1/2	Inhibition	Protective autophagy	*In vitro*: U87, C6, and U251*In vivo*: C6	(230)
TMZ + Irradiation	Increase of Beclin-1, ATG5	Activation	Lethal autophagy	*In vitro*: T98 and U373	(175)
TMZ + THC + CBD	Induction of autophagy-associated apoptosis and toxicity	Activation	Lethal autophagy	*In vivo*: U87	(237)
TMZ + CA	Induction cell cycle arrest and apoptosis, inhibition of p-AKT	Activation	–	*In vitro*: U251 and LN229	(238)
TMZ + GDC-0941	Induction of cell cycle arrest and apoptosis, inhibition of p-AKT and MGMT	Activation	–	*In vitro*: A172, T98, and SHG44	(239)
TMZ + MTB	Induction of apoptosis, inhibition of JAK2/STAT3	Activation	–	In vivo: U251	(240)
CQ + Galunisertib	Inhibition of TGF-β2-induced autophagy	Inhibition	Protective autophagy	*In vitro*: U87, T98, and U251 *In vivo*: U87	(241)
CQ + BAFA1	ROS induction	Inhibition	Protective autophagy	*In vitro*: U87 and C6	(179)
2DG + CP	ER stress induction, induction of apoptosis	Inhibition	Protective autophagy	*In vitro*:LN229 and A172	(242)
IM + TIC	Induction of non-apoptosis cell death *via* AC/cAMP/EPAC1 signaling pathway	Activation	Lethal autophagy	*In vitro*: LN71, LN229, and LN443 *In vivo*: LN229	(158)
Erlotinib + Sorafenib	Inhibition AKT and ERK signaling	Activation	Lethal autophagy	*In vitro*: U87, LNZ308, LN428, and GSC	(243)

TMZ, temozolomide; CQ, chloroquine; HCQ, hydroxychloroquine; SAHA, suberoylanilide hydroxamic acid; THC, delta-9-Tetrahydrocannabinol; CBD, cannabidiol; CA, carnosic acid; MTB, momelotinib; BAFA1, bafilomycin A1; 2DG, 2-deoxy-D-glucose; CP, cisplatin; IM, imipramine; TIC, ticlopidin.

Apart from inhibiting protective autophagy, another direction may involve the agents triggering cytotoxic autophagy. Delta-9-tetrahydrocannabinol (THC) and cannabidiol (CBD) are both cannabinoids utilized as anticancer agents due to their ability to induce lethal autophagy (244). The systemic administration of THC + CBD at a 1:1 ratio in combination with TMZ strongly reduces the subcutaneous and the intracranial tumor volume in the preclinical models of glioma, which results in complete tumor regression in over half of the animals (237). Indeed, the TMZ/THC/CBD therapy has already been proposed to be evaluated through a clinical trial (NCT03529448). Although microRNAs are mostly regarded as biomarkers for diagnosis or prognosis, the synergistic effect of miR-450a-5p overexpression combined with epidermal growth factor receptor (EGFR) inhibitor gefitinib confers the sensitivity to chemotherapy in glioma through the EGFR-stimulated PI3K/AKT/mTOR signaling pathway and further pro-death autophagy activation (245). Furthermore, the application of rapamycin induces autophagy and results in an increase of radiosensitivity in EGFR-silencing GBM cell lines (246).

With the addition of carnosic acid (CA), an abietane diterpenoid extracted from rosemary or common sage, the U251 and LN229 cells are resensitized to TMZ incubation showing Cyclin B1-mediated cell cycle arrest, cellular apoptosis induction by cleavage of PARP and Caspase-3, and enhanced autophagy through inhibition of phosphorylated AKT (238). This is similar to how pan-PI3K inhibitor GDC-0941 combined with TMZ arrests cell cycle, exerts a pro-apoptotic effect, and augments autophagy mainly through the PI3K-AKT signaling pathway (239). The Janus kinase (JAK) inhibitor momelotinib (MTB) also potentiates TMZ efficacy *via* apoptosis and autophagy induction (240). Although TMZ/CA, the TMZ/GDC-0941, and the TMZ/MTB therapy fail to identify the relation between the relationship between apoptosis and autophagy, it is plausible to think that the “apoptotic” cell death could be ascribed to autophagy-associated impacts. Some of the autophagy-related agents treating glioma are summarized in **Table 1**, while the other autophagy modulating chemicals or autophagy-related molecular targets have been discussed elsewhere (181, 247, 248).

## Conclusion and Future Perspective

Autophagy represents not merely a way for cellular maintenance, but also a key point of regulation in finely adjusting interplays between glioma and the TIME. It supports glioma typically by enhancing the adaptability of glioma cells to the extent of progressive, invasive, and drug-resistant malignancy, and also by rendering prevailing TAMs differentiating towards the M2 phenotype through exosomes and reduce phagocytosis. Neutrophils use autophagy for proper immunity against glioma. The intercellular engagement, activation, and anti-glioma activities of T cells are ensured with autophagy involvement.

Conclusively, the ultimate effect of autophagy in glioma and the TIME may highly depend on the cell type, the intensity of the surrounding signal, and the stage of the lesion and progression. Autophagy can be utilized to cater to a variety of demands, but it seemingly turned out to be tumor-promoting cumulatively with glioma progression. It also should be reiterated that autophagy may influence the status and well-being of glioma cells and the members of TIME, and thereby modulate their interconnections in the permissive environment. Some interplays may rely on the whole process of autophagy while others are involved with some of the autophagic components instead of the entire flux.

Beyond glioma cells, autophagy in any peritumor cells would be influenced when autophagy is manipulated systemically. Considering the promising efficacy of autophagy modulators and the definite number of related clinical trials and basic studies, it is of great importance to shed more light on the entire supervision of glioma cells and their environment at a single-cell level, of which the members in the suppressive immune environment should be prioritized. Therefore, more profound insights will be acquired for novel autophagy-related therapy development.

The reason why it deserves more attention not only comes from the trend that autophagy modulators might be part of regular use for glioma treatment one day but also lies in the autophagy-related drugs that are being clinically employed. However, the mechanism challenges regarding autophagy in glioma and its TIME are pressing. How autophagy contributes to oncosuppression and turns itself into an indispensable part of glioma malignancy remains to be determined. It is also critical to identify specific and easily-tested indicators for autophagy status so that the dependency on autophagy for a certain type of cells in glioma is characterized before autophagy therapies.

## Author Contributions

The study and conception were designed by YF, CM, and GZ. The manuscript was drifted by YF, YW, JZ, XD, PG, and KL. Figures were prepared by YF, YW, JZ, and XD. Reviewing and editing was completed by CM and GZ. All authors contributed to the article and approved the submitted version.

## Funding

We acknowledge the funding support from National Nature and Science Foundation of China (Grant Number: 81772684 and 81872050), the S&T Development Planning Program of Jilin Province (Grant Number: 20200201469JC, 20200201613JC, and 20200201388JC), and Foundation from the Development and Reform Commission of Jilin Province (Grant Number: 2017C059-2, and Chinese People's Brain Neural Network Research and Innovation Cooperation Platform Construction Project).

## Conflict of Interest

The authors declare that the research was conducted in the absence of any commercial or financial relationships that could be construed as a potential conflict of interest.

## Publisher’s Note

All claims expressed in this article are solely those of the authors and do not necessarily represent those of their affiliated organizations, or those of the publisher, the editors and the reviewers. Any product that may be evaluated in this article, or claim that may be made by its manufacturer, is not guaranteed or endorsed by the publisher.
